# Characterization of Ingested Microplastics in a Regional Endemic Lizard *Apathya cappadocica* (Werner, 1902) from Türkiye

**DOI:** 10.3390/biology14101457

**Published:** 2025-10-21

**Authors:** Cantekin Dursun, Nagihan Demirci, Kamil Candan, Ahmet Gökay Korkmaz, Ecem Büşra Hastürk, Elif Yıldırım Caynak, Çetin Ilgaz, Yusuf Kumlutaş, Serkan Gül

**Affiliations:** 1 Department of Biology, Faculty of Arts and Sciences, Recep Tayyip Erdoğan University, 53100 Rize, Turkey; cantekin.dursun@erdogan.edu.tr (C.D.); nagihan_demirci19@erdogan.edu.tr (N.D.); 2 Department of Biology, Faculty of Science, Dokuz Eylül University, Buca, 35390 İzmir, Turkey; kamil.candan@deu.edu.tr (K.C.); ahmetgokay.korkmaz@deu.edu.tr (A.G.K.); hasturk.ecembusra@ogr.deu.edu.tr (E.B.H.); yildirim.elif@deu.edu.tr (E.Y.C.); cetin.ilgaz@deu.edu.tr (Ç.I.); yusuf.kumlutas@deu.edu.tr (Y.K.); 3 Fauna and Flora Research and Application Center, Dokuz Eylül University, Buca, 35390 İzmir, Turkey

**Keywords:** bioaccumulate, ecosystem, food web, reptile

## Abstract

This study investigated the presence, characteristics, and distribution of ingested microplastics (MPs) in *Apathya cappadocica*, a regional endemic lizard species. A total of 93 individuals were examined, and MPs were found in 19.35% (n = 18) of them. A total of 27 microplastic particles were detected, averaging 1.5 MPs per positive individual and 0.29 MPs per individual across the sample. MP sizes ranged from 50 to 1727 µm, with a mean size of 355.46 ± 73 µm. Most MPs (93%) were fibers, while the rest were fragments. The dominant color was navy blue (41%), followed by red and black (19% each). Polyvinyl alcohol (PVA) was the most common polymer (67%), followed by polyethylene terephthalate (PET) and polyethylene (PE). Statistical tests revealed no significant differences in MP size based on shape, color, or type, nor any correlation between MP size and gastrointestinal tract weight. However, microplastic shape was significantly associated with polymer type; fiber MPs consisted mainly of PET and PVA, while fragments were equally split between PVA and PE. These findings indicate that terrestrial reptiles are exposed to microplastic pollution and that microplastic characteristics may provide insights into their potential environmental sources.

## Simple Summary

This study investigated the presence of microplastics (MPs) in the digestive systems of individuals of *Apathya cappadocica*, a regional endemic lizard species. Microplastic particles were detected in approximately 19% of the 93 individuals examined. Most of these particles were fibrous, with the most common color being dark blue and the most common plastic type being polyvinyl alcohol (PVA). Statistical analyses revealed that microplastic size did not significantly differ based on shape, color, or type. However, a significant relationship was found between the shape of the microplastics and the type of plastic. These results indicate that reptiles living in terrestrial environments are also affected by microplastic pollution and provide clues about the sources of plastic in the environment.

## 1. Introduction

In recent years, an increase in plastic production and its resulting environmental dispersion have caused serious ecological problems in both aquatic and terrestrial habitats. Microplastics (MPs), formed as a result of the breakdown of plastics over time by physical, chemical, and biological agents, are persistent pollutants with sizes below five mm that can easily spread in the environment [1]. Initially reported extensively in marine systems, MPs now pose a significant pollution threat to terrestrial ecosystems as well [2,3]. Their long-term persistence in soil, their ability to combine with pesticides, and their easy interaction with biota further increase the environmental risk potential of these pollutants [4,5]. MPs enter the environment from two main sources: primary and secondary. Primary microplastics (e.g., microbeads in cosmetics, fibers from the washing of synthetic textiles, and particles from tire abrasion) are released directly into the environment, while secondary microplastics are formed over time through the breakdown of larger plastic waste (e.g., bags, bottles) through physical, chemical, and biological processes [6]. These different sources directly influence the distribution of microplastics across marine, freshwater, and terrestrial ecosystems, leading to a global pollution problem [7]. MPs can be ingested by many organisms, especially invertebrates, mistaking them for food, and can bioaccumulate in the digestive tract [8]. Smaller particles, in particular, have the potential to cross the digestive barrier and enter tissues or the bloodstream. These particles, which accumulate in organisms at lower levels of the food chain, can be transferred to higher trophic levels through predator–prey interactions, causing biomagnification. This poses serious risks to organisms at the top of the food chain [9]. While most studies on the effects of MPs on biological organisms have focused on marine vertebrates and birds, terrestrial vertebrate groups, especially reptiles, have been largely neglected [10,11]. However, reptiles may be directly exposed to microplastics due to their generally low-lying feeding behavior, their presence in both urban and natural habitats, and their proximity to agricultural lands [12,13,14]. The presence of microplastics in reptiles is mostly revealed through digestive system analyses, and advanced characterization techniques such as Fourier Transform Infrared Spectroscopy (FTIR) provide information on particle shape, color, size, and polymer structure [15,16,17]. Such analyses provide important clues not only to biological exposure but also to the environmental source and potential toxicological effects of microplastics [18,19]. Türkiye has a rich biodiversity, hosting diverse climates and habitat types. However, studies addressing the impacts of microplastics on native and endemic species are quite limited [20,21].

The environmental hazards of MPs involve both physical and chemical mechanisms. Physically, they can cause blockages in the digestive tract, leading to a false sense of fullness and reduced food intake. Chemically, the high surface area of MPs allows them to readily adsorb hydrophobic contaminants (e.g., pesticides, heavy metals, PCBs) in water and soil [22]. When these contaminated particles are ingested by organisms, these toxic substances can be transferred into the body, leading to harmful biological effects [23]. In this context, our hypothesis is to test whether *Apathya cappadocica*, a species living in terrestrial habitats, may exhibit microplastic accumulation due to its feeding habits and environmental exposure. For this, our aims to (1) to determine the presence, frequency, and distribution of microplastics in the digestive tract of *A. cappadocica*, (2) to analyze the morphological (shape, size, color) and chemical (polymer type) properties of the detected microplastics, and (3) to evaluate the findings in the context of the species’ habitat and feeding ecology and to relate them to potential environmental sources. While microplastic research in Türkiye has generally focused on marine systems, studies on the exposure of endemic and terrestrial vertebrate species to microplastic pollution are limited [14,20,21]. This study is particularly original as it is the first to be conducted on *A. cappadocica*, a regionally endemic reptile species, and provides important data on microplastic pollution in terrestrial ecosystems. Furthermore, it will not only determine the species’ exposure level but also fill a significant gap in the literature regarding the effects of terrestrial microplastic pollution on reptiles.

## 2. Materials and Methods

### 2.1. Sampling

**Figure 1:**
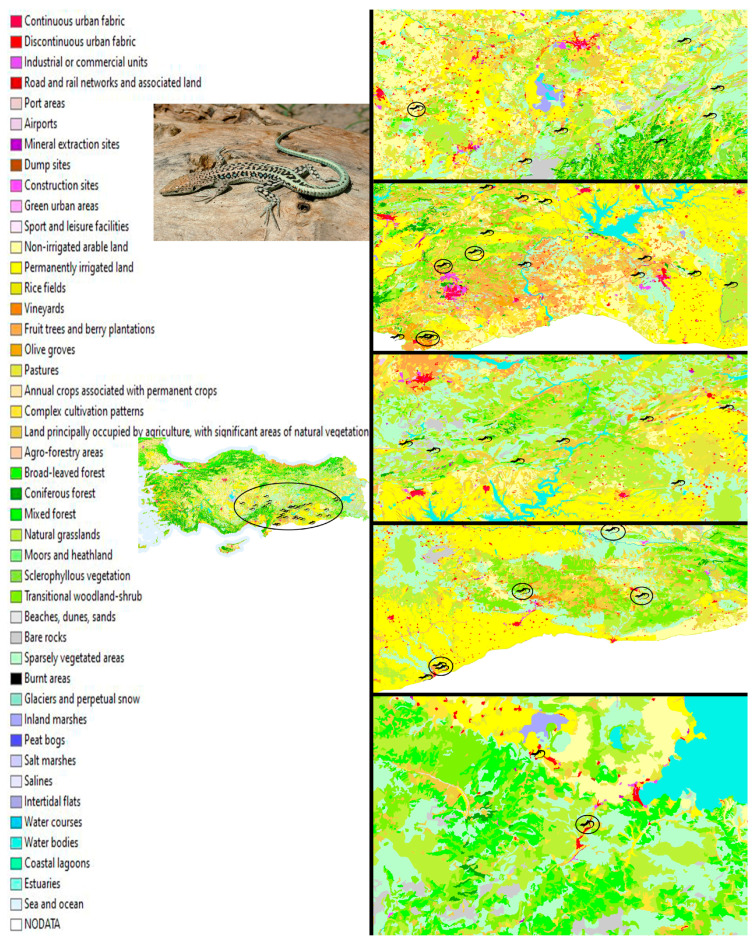
The map shows land cover and land cover changes for Türkiye between 1990 and 2018 (adapted from [27] as open and free access), with symbols indicating the known distribution sites of *Apathya cappadocica*. The circle symbols present localities where microplastics have been found.

The distribution range of *Apathya cappadocica* includes Central, Eastern, and Southeastern Anatolia in Türkiye, as well as parts of Northern Syria, Northern Iraq, and Northwestern Iran [24] (Figure 1). A total of 93 adult specimens of *Apathya cappadocica* were provided from the museum collections of the Fauna and Flora Research and Application Center at Dokuz Eylül University. Samples collected by hand between 1993 and 2006 were previously preserved in glass jars filled with 96% ethanol. For subsequent microplastic analysis, gastrointestinal tracts (GITs) were carefully removed in their entirety by severing them at both the upper esophagus and the anal orifice. For this process, steel-made scalpels, scissors, and trays were used for the removal process. The precise weight of each individual gastrointestinal tract was determined to the nearest 0.01 g using an analytical balance Ohaus EX223N (Dundas, ON, Canada). All samples were then stored in new glass jars with 96% ethanol (Merck, Darmstadt, Germany), ensuring their preservation for further investigation. Museum collections are an indispensable resource for such retrospective analyses [25]. Ethanol is a widely accepted method for long-term preservation of biological specimens. Studies show that it does not chemically react with the most common polymers and does not degrade the structure of microplastics [26]. Collecting samples from different geographical regions of Türkiye reduces the risk of local bias. Furthermore, preserving the samples in glass jars and 96% ethanol increases the reliability of the results.

### 2.2. Microplastic Characterization

**Figure 2:**
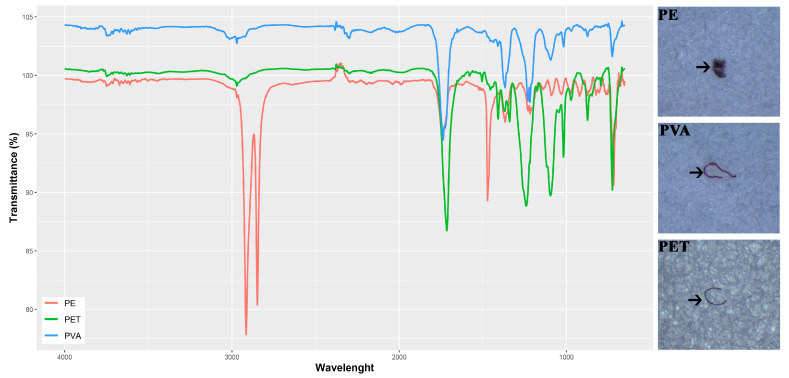
FTIR peaks of three different MP types were determined in this study.

MPs were extracted from the gastrointestinal tissues of adult lizards through chemical digestion using a potent oxidizing agent—30% hydrogen peroxide (H_2_O_2_, *w*/*w*) (Tekkim Ltd. Com. Bursa, Türkiye). For the digestion process, pre-weighed gastrointestinal tracts were placed into glass tubes (25 cm long, 2.5 cm in diameter). These tubes were set inside a batch reactor operating at a constant temperature of 65 °C. Tissues were treated with 5 mL of H_2_O_2_ solution. To reduce both evaporation and potential contamination, the tubes were covered with watch glasses during the digestion phase. After the tissues were fully digested, the remaining material was filtered under vacuum using Whatman Grade 4 qualitative filter paper, which has a pore size of 20–25 µm. The filters were then placed in glass Petri dishes for microscopic examination. Potential microplastic particles were visually identified with a Leica S6D^®^ stereomicroscope (Wetzlar, Germany) based on their shape and coloration. During the analysis, we targeted the most commonly found polymer types in environmental samples. These include polymers such as polyethylene (PE), polypropylene (PP), polyvinyl chloride (PVC), polyethylene terephthalate (PET), and polyvinyl alcohol (PVA). To confirm polymer composition, FT–IR was applied using a PerkinElmer Spectrum 100 spectrometer, equipped with an attenuated total reflectance (ATR) module (PerkinElmer, Waltham, MA, USA). Spectra were recorded across the 4000–650 cm^−1^ range, using 12 scans per particle at a resolution of 2 cm^−1^. Each spectrum was compared against entries in the PerkinElmer SEARCH Plus ATR Polymer database. Particles were identified as microplastics if the spectral match was at least 70% as shown in (Figure 2) (Supplementary Materials Figures S1–S27). This approach allowed us to clearly define the scope and objectives of our methodology by focusing only on the most common polymers. A foldable magnifying glass (8×) was used to carefully carry and place MPs from the Petri dish to FT–IR. To make it clear how well our workflow can identify things, it is important to remember that the Whatman Grade 4 filter paper has a nominal pore size of 20–25 μm, which is what the manufacturer says it should be able to become under normal conditions. In actuality, retention efficiency is influenced by particle shape, filter construction, and vacuum pressure, and so does not exactly correlate with the smallest reliably detectable particle in subsequent analysis. We used a Leica S6D^®^ stereomicroscope for visual identification in our investigation. This microscope is best for particles that are tens to hundreds of micrometers in size. Additionally, ATR–FTIR polymer validation needs a large enough surface contact area to make high-quality spectra, which means that the practical lower detection threshold of our combined approach is bigger than the theoretical pore size. As a result, the smallest particle size that could be successfully detected and validated in our dataset was about 50 μm. Microplastics were measured using ImageJ software v1.46r [28]. A calibrated reference scale (1 mm at 4× magnification) was used to determine particle lengths (Supplementary Materials Table S1). Throughout the entire procedure, a rigorous protocol was implemented to minimize and monitor potential microplastic contamination. All equipment and glassware were thoroughly rinsed with triple-distilled water before use. In addition to the use of 100% cotton lab coats and nitrile gloves, three types of blank samples were included as follows: (1) to assess contamination from chemicals, a negative control was performed by processing a mixture of 5 mL of 30% H_2_O_2_ and 96% ethanol without any tissue sample, (2) to monitor airborne contamination, a glass Petri dish containing a filter paper was left exposed in the laboratory for the duration of the sample processing time, and (3) to account for potential contamination during sample handling, a glass dish was subjected to all procedural steps—including the use of scalpels and scissors—without any sample. All blank samples were processed and examined identically to the study samples using FTIR. No microplastic particles were detected in any of the blank samples.

### 2.3. Statistical Process

Descriptive statistics related to microplastic characteristics were calculated using the *psych* package [29]. Tests for normality were conducted using the *olssr* package [30]. Differences in microplastic sizes across various MP patterns were assessed using the Kruskal–Wallis and Wilcoxon Rank Sum tests. Categorical variables, including microplastic type, color, and shape, were analyzed using Pearson’s chi-square test based on frequency distributions with a Type 2 error. Correlations between continuous variables were also examined to test linear relationships. All analyses were conducted using the *stats* package [31]. To illustrate the proportion of each microplastic characteristic, doughnut charts were generated using the *lessR* package [32]. Stacked bar plots were employed to visualize the distribution of subcategories within microplastic features. The distribution of microplastic sizes across types was represented using box plots, histograms, and density plots. Additionally, a scatter plot was created to depict the relationship between microplastic size and gastrointestinal tract weight. All visualizations of microplastic data were produced using the *ggplot2* package [33]. Analyses and visualizations were carried out in R Programming Language v4.5 [31].

## 3. Results

MPs were detected in 18 out of 93 individuals, corresponding to 19.35% of the total samples. The mean SVL was 6.79 ± 0.07 cm and ranged between 5.2 and 8.3 cm. The mean weight was 8.58 ± 0.32 g and ranged between 4 and 18.6 g. For MP-positive individuals, SVL was 7.24 ± 0.16 cm, whereas the mean weight was 9.36 ± 0.89 g. A total of 27 microplastic items were identified, with an average of 1.5 items per microplastic positive individual and 0.29 items per individual across the entire dataset. A maximum of three microplastic items were found in three individuals. Three individuals contained two microplastic items, while the remaining had one item each. The average size of MPs was 355.46 ± 73 µm, ranging from 50 µm to 1727 µm. Descriptive statistics are provided in Table 1.

**Figure 3:**
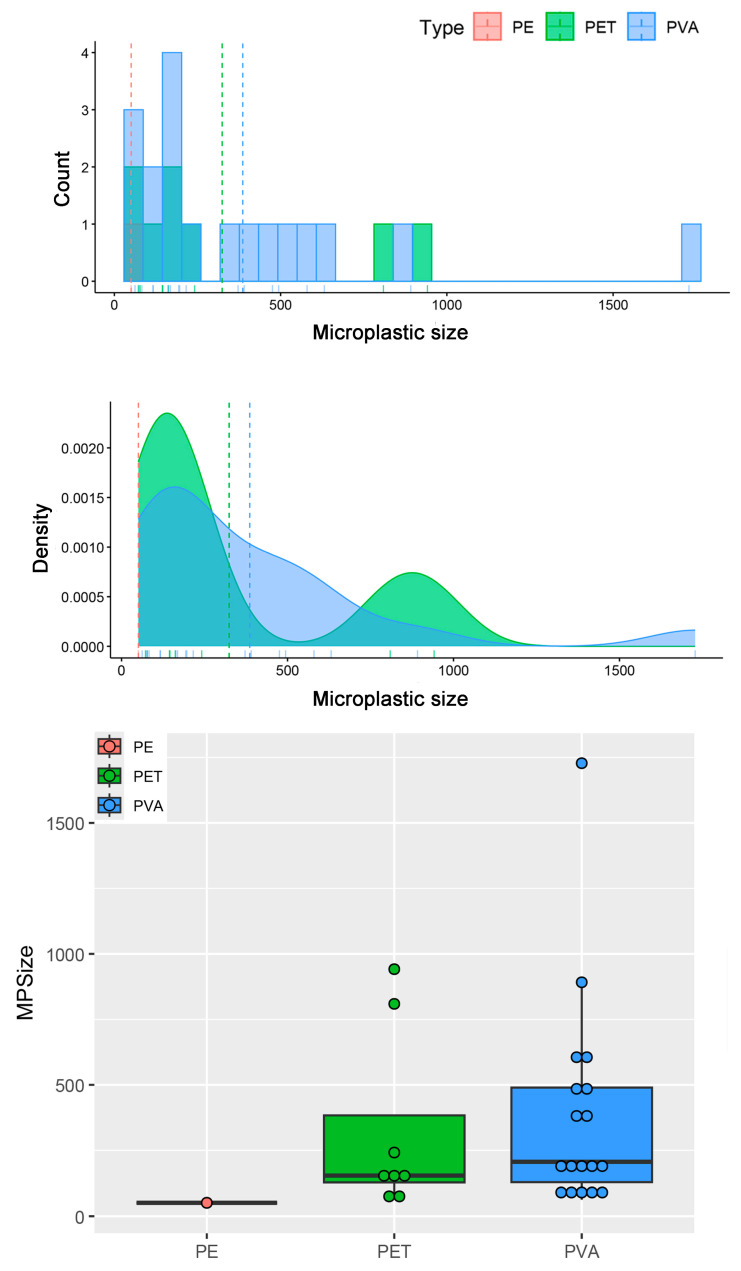
Size distribution of microplastic types represented with histograms, density, and boxplots. Vertical dashed lines in histogram graphics demonstrate the mean microplastic size. Dark lines in boxes show the median, and each dot represents an observed microplastic item.

According to the Shapiro–Wilk test, the size data did not follow a normal distribution (*p* < 0.001). The Kruskal–Wallis test showed no significant differences in microplastic size across types (χ^2^ = 3.13; df = 2; *p* > 0.05) or colors (χ^2^ = 10.35; df = 7; *p* > 0.05). The longest microplastic fragment was found in polyvinyl alcohol (PVA), while the shortest was in polyethylene (PE), as shown in (Figure 3).

**Figure 4:**
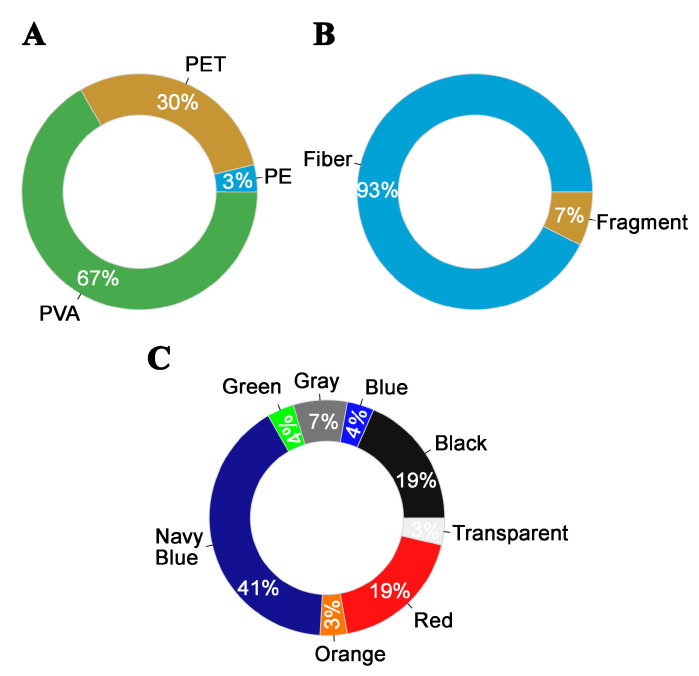
Doughnut graphics demonstrate the percentage of microplastic patterns in each category. (**A**) Type; (**B**) Shape; (**C**) Color.

PVA was the most frequently observed plastic type, accounting for 67% of all MPs. Navy blue was the dominant color (41%), followed by red and black, each comprising 19%. In terms of shape, 93% of MPs were fibers, and the remainder were fragments, as shown in (Figure 4).

**Figure 5:**
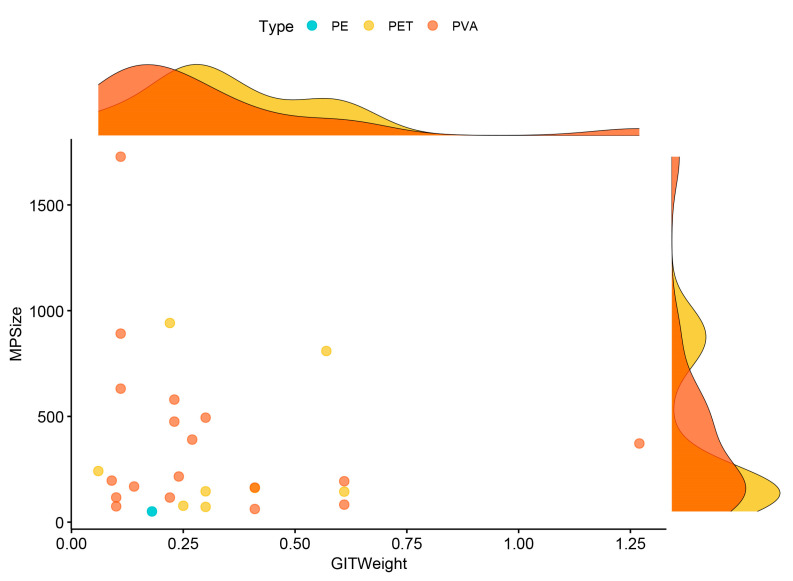
A scatterplot representing the relationship between microplastic size and gastrointestinal tract weight. Each dot represents a single microplastic item. Observations were colored based on microplastic types. Density plots show the distribution of size and weight data with microplastic types.

No significant size difference was observed between microplastic shapes (W = 46; *p* > 0.05). Similarly, gastrointestinal tract weights of microplastic-positive individuals showed no significant differences based on microplastic type (χ^2^ = 1.34; df = 2; *p* > 0.05), color (χ^2^ = 3.99; df = 7; *p* > 0.05), or shape (W = 19; *p* > 0.05). A statistically significant relationship was found between SVL and weight (r = 0.59; *p* < 0.01), SVL and gastrointestinal tract weight (r = 0.51; *p* < 0.01), but SVL and microplastic size (r = −0.19; *p* > 0.05), weight and microplastic size (r = −0.19; *p* > 0.05), weight and gastrointestinal tract weight (r = 0.26; *p* > 0.05), and microplastic size and gastrointestinal tract weight (r = −0.13; *p* > 0.05, Figure 5). For all samples (N = 93), there was no correlation between SVL and microplastic size (r = −0.16 *p* > 0.05), weight and microplastic size (r = −0.31; *p* > 0.05), weight and gastrointestinal tract weight (r = 0.26; *p* > 0.05), and microplastic size and gastrointestinal tract weight (r = −0.14; *p* > 0.05), but SVL and weight (r = 0.73, *p* < 0.01), and weight and GIT weight (r = 0.45, *p* < 0.01).

**Figure 6:**
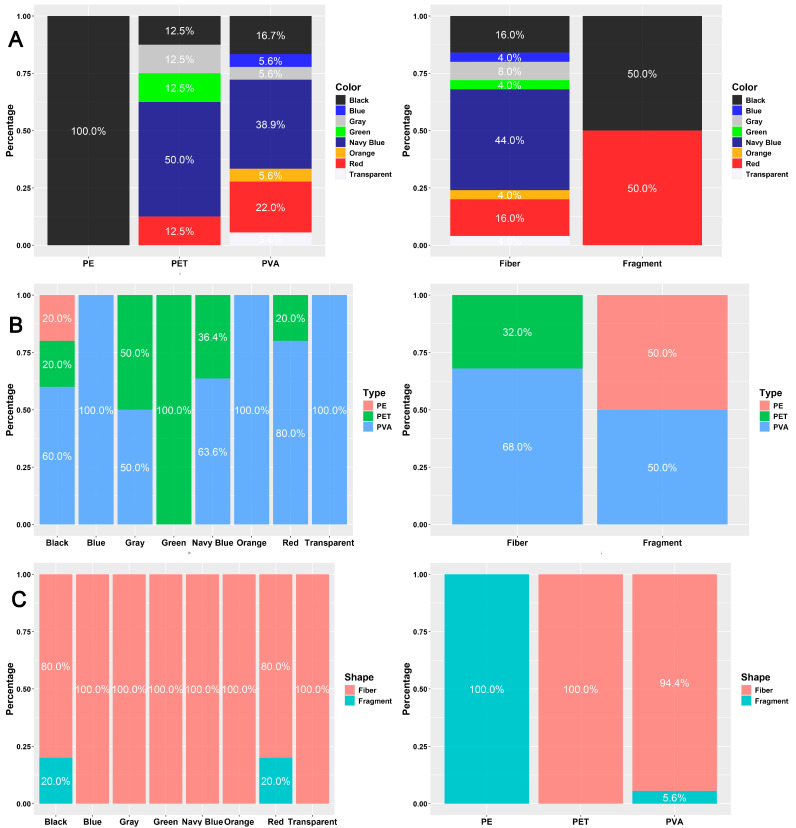
Stacked bar charts displaying the relative abundance of microplastic characteristics between different categories. (**A**) Color; (**B**) Type; (**C**) Shape.

The number of microplastic items did not significantly differ when classified by color and type (χ^2^ = 9.15; df = 14; *p* > 0.05) or by color and shape (χ^2^ = 3.67; df = 7; *p* > 0.05). Among the color categories, black MPs comprised three different types. Transparent, orange, and blue items were exclusively PVA, while the single green item was polyethylene terephthalate (PET), as shown in (Figure 6). Red and black MPs included both fragments and fibers, whereas other colors consisted solely of fibers. PVA items were found in seven different colors, PET in five, and the single PE item was a black fragment. All PET items were fibers. A significant association was found between microplastic shape and type (χ^2^ = 13.23; df = 2; *p* < 0.01). Fiber MPs were primarily composed of PET (68%) and PVA (32%), while fragments were evenly split between PVA and PE (50% each).

## 4. Discussion

Microplastic contamination has emerged as a pervasive global pollutant, with documented impacts across a wide range of ecosystems and organisms. Marine environments, in particular, have received significant attention, where numerous studies have highlighted MP accumulation in fish [34,35,36]. In freshwater habitats, both fish and amphibians became subjects of MP monitoring [37,38,39,40,41]. As for reptiles, despite their ecological significance and vulnerability, they have been comparatively overlooked in this context. Among them, turtles from marine and freshwater habitats have been more frequently assessed, revealing frequent ingestion and exposure [15,42,43,44]. However, terrestrial and freshwater reptile species remain largely underrepresented in MP research, creating a substantial gap in understanding [12,13,14,21]. This disparity underscores the need to broaden the scope of studies to include less-explored reptilian taxa and habitats, particularly given the potential for widespread exposure across trophic levels and ecosystems.

In this study, the mean MP size was found to be 355.46 ± 73.26 µm. Dursun et al. [14] traced MP presence in a terrestrial lizard, *Ophisops elegans*, from Türkiye. They examined 300 individuals, and MP items were found in only 25 samples, regarding 8.33%. The number of detected MP items was 37, and the mean size was 285.59  ±  57.72 µm. The number of MPs per sample was 1.48, whereas it was 0.12 for all samples. Another study on microplastic pollution was conducted with *Blanus strauchi*, a limbless lizard living in subterranean habitats [21]. MPs were unveiled in the GITs of 29 specimens, regarding 24.57% of all samples (N = 118). In total, 34 MP items were identified, with an average of 1.17 particles per individual and 0.28 particles per individual for all examined samples. The overall mean length of MPs was calculated to be 969.47 ± 144.37 µm.

Mackenzie and Vladimirova [45] found MPs in 12.03% of 133 fecal samples from *Hemidactylus mabouia* and in 6.00% of 50 samples from the *Tropidurus torquatus* representing Paraguay herpetofauna. The average number of MPs per sample was 0.12 and 0.06. In contrast, Teampanpong and Duengkae [13] detected MPs in five out of six distinct lizard species (71.43%) in western Thailand, with an average of 1.29 MP particles per individual. Additionally, the presence of MPs in the feces of the Butterfly lizard *Leiolepis belliana* was reported as 1.33 items per sample [46].

Given the existing literature, the number of items per MP positive individuals in this study was higher than the reported studies above. On the other hand, Lu et al. [47] reported the number of MP items per individual as 4.13 for *Gekko subpalmatus*. From that aspect, it can be thought that these differences are more related to anthropogenic pressure in habitats in which the individuals are sampled. The mean MP size reported for *Blanus strauchi* was almost three times larger than the finding in this study. It can be hypothesized that the GIT of the Turkish worm lizard has an elongated shape, and the digestive system extends along the body, allowing it to ingest larger MP items.

Even though a limited number of studies presented above assessed MP contamination in terrestrial reptiles, fibers consistently emerge as the most common type of microplastic detected. Dursun et al. [14] reported that 97.29% of MPs were fiber observed in the gastrointestinal tract of *Ophisops elegans*. In the study of Dursun et al. [21], all MPs were determined as fiber (100%) for *Blanus strauchi*. The fiber rate also was found to be 84.84% in Mackenzie and Vladimirova [45], 77% in Teampanpong and Duengkae [13], 95.18% in Teampanpong and Duengkae [46], and 94.70% in Gül et al. [20]. Fiber microplastics are widely observed in MP contamination investigations, primarily originating from synthetic textiles and the breakdown of fibrous plastic materials. They can penetrate into the different environments through domestic washing, industrial processes, atmospheric deposition, and degradation of larger plastic items [48,49,50]. Due to their lightweight and elongated shape, fibers are easily transported by natural factors such as wind and water. Therefore, they are frequently detected in animals [51,52,53]. For terrestrial reptiles, fibers may be ingested unintentionally through contaminated prey such as insects. Furthermore, land use in our sampling areas may lead to different types of microplastic exposure because individuals living in habitats close to urban areas and roadsides are likely to be more frequently exposed to fiber-type MPs originating in areas with high urbanization and human activity. These microplastics can typically originate from wastewater generated by the washing of synthetic textiles or plastic waste generated by the breakdown of packaging materials.

Polyvinyl alcohol (PVA) comprised more than half of the detected MP items in this study. It is a water-soluble synthetic polymer generally used in various industrial and consumer products, including packaging films, textiles, and adhesives. This could indicate a regional pollution source related to environmental factors such as local industrial activities or agricultural practices. Furthermore, this finding demonstrates that even PVA, considered biodegradable, can persist in natural ecosystems and be ingested by living organisms. This unique finding reflects not only the pollution in this species but also the regional pollution profile of the investigated region. PVA can enter natural environments, especially with industrial wastewater disposal or breakdown of larger materials. Although it is known as biodegradable, the process can occur slowly, causing the permanence of the materials in natural habitats. PET (polyethylene terephthalate) was the second abundant polymer type extremely used in industrial production regarding plastic water bottles, textile manufacturing, and food packs. It occurs by fragmentation of larger plastic, and the material is highly durable and resistant to degradation, allowing it to persist in ecosystems for long periods.

PVA is rarely reported in previous studies of reptiles. Gül et al. [20] recorded that 5.30% of the MPs for *Natrix natrix* and 11.80% for *Natrix tessellata* were PVA across Türkiye. Dursun et al. [21] reported PVA presence in *Blanus strauchi* as 3.00%. However, PET was the most abundant polymer in both studies, noted 68.40% for *N. natrix*, 41.20% for *N. tessellata*, and 94.12% for *B. strauchi*. For the lizard *O. elegans* in the study of Dursun et al. [14], a total of seven different plastic types were characterized, and the rate of PET was 54.06%. Considering the abundance and diversity of different organisms, it can be proposed that the source of MP pollution and organismal behavior, such as foraging or diet preference, can affect ingested MPs because the feeding habits vary significantly among herpetofauna species. Many lizards consume a large portion of their diet consisting of insects and other invertebrates. This prey may be the primary carriers of MPs. Comparative studies suggest that the specific prey preferences of different species may influence the type, size, and quantity of ingested MPs. For example, the diet of subterranean-dwelling *Blanus strauchi* consists of soil-dwelling earthworms and insect larvae, while surface-dwelling species such as *Apathya cappadocica* and *Ophisops elegans* have more diverse prey. This may lead to differences in the source and physical characteristics (size, shape) of ingested MPs.

As for color, navy blue, red, and black items were prevalent in observed MPs. Dursun et al. [14] determined that the main colors were blue (48.65%) and red (24.33%), while Dursun et al. [21] identified the most abundant colors as blue (38.23%) and black (26.47%), as in Gül et al. [20]. Although Teampanpong and Duengkae [13] handled five different animal groups, including reptiles, the dominant colors were blue (41.86%) and black (19.64%). From that aspect, it can be assumed that the three main colors—blue, black, and red—may be characteristic of microplastics ingested by reptiles. Especially, blue items, predominantly originating from synthetic materials used in textiles and disposable water bottles, tend to degrade faster under sunlight, making them more bioavailable in the environment. Due to anthropogenic debris accumulation in terrestrial and aquatic habitats, it can be reasonable to observe blue MPs often in reptile digestive systems.

## 5. Conclusions

This study provides a valuable contribution to the growing, yet still limited, body of research on microplastic pollution in terrestrial reptiles. By focusing on a single species, *Apathya cappadocica*, we revealed species-specific patterns in microplastic (MP) characteristics such as shape, size, and polymer types. Our findings confirm that the observed MP characteristics are largely consistent with previous studies but suggest that variation in MP characteristics and different ratios may be more related to the species’ habitat-specific use or feeding ecology. Our data demonstrates the exposure of terrestrial reptiles to microplastic pollution. However, more comprehensive studies are urgently needed to draw broader conclusions across all reptile groups. These results highlight the importance of broader monitoring efforts targeting diverse reptile groups and habitats to identify new conservation strategies. Also, an important methodological limitation concerns the underrepresentation of very small microplastics (<50 μm). Although the filters used theoretically retain particles down to 20–25 μm, our workflow—based on stereomicroscopic visual sorting and ATR–FTIR confirmation—makes reliable detection below ~50 μm challenging. Smaller particles are more difficult to identify visually, may fail to produce interpretable FTIR spectra, and can be partially lost during filtration or transfer steps. As a result, our dataset should be considered a conservative estimate of microplastic abundance, particularly with respect to the smallest size fractions and nano plastics. We recommend that future studies apply advanced analytical approaches such as µ-FTIR or Raman imaging, together with recovery experiments, to capture this size range fully.

## Figures and Tables

**Table 1:** Descriptive statistics of identified MPs in different patterns.

		MP Size (μm)		GIT Weight (g)	
	N	Mean ± SE	Min–Max	Mean ± SE	Min–Max
All items	27	355.46 ± 73.26	50.53–1727.97	0.31 ± 0.05	0.06–1.27
Type	N	Mean ± SE	Min–Max	Mean ± SE	Min–Max
PE	1	50.53	-	0.18	-
PET	8	324.19 ± 122.35	71.96–941.59	0.34 ± 0.06	0.06–0.61
PVA	18	386.31 ± 95.86	62.09–1727.97	0.31 ± 0.07	0.09–1.27
Color		Mean ± SE	Min–Max	Mean ± SE	Min–Max
Black	5	92.27 ± 26.51	50.53–196.57	0.21 ± 0.06	0.09–0.41
Blue	1	193.44	-	0.61	-
Gray	2	318.59 ± 156.92	161.67–475.51	0.32 ± 0.09	0.23–0.41
Green	1	941.59	-	0.22	-
Navy Blue	11	298.04 ± 70.95	71.96–809.19	0.35 ± 0.11	0.06–1.27
Orange	1	494.21	-	0.30	-
Red	5	602.33 ± 317.94	82.74–1727.97	0.31 ± 0.09	0.1–0.61
Transparent	1	579.65	-	0.23	-
Shape		Mean ± SE	Min–Max	Mean ± SE	Min–Max
Fiber	25	378.57 ± 77.32	62.09–1727.97	0.31 ± 0.05	0.06– 1.27
Fragment	2	66.63 ± 16.10	50.53–82.74	0.40 ± 0.21	0.18–0.61

## Data Availability

The original contributions presented in this study are included in the article. Further inquiries can be directed to the corresponding authors.

## Supplementary Materials

The following supporting information can be downloaded at: https://www.mdpi.com/article/10.3390/biology14101457/s1, Figures S1–S27; Table S1.
